# Feasibility and safety of choledochotomy primary closure in laparoscopic common bile duct exploration without biliary drainage: a retrospective study

**DOI:** 10.1038/s41598-023-49173-3

**Published:** 2023-12-18

**Authors:** Wei Lai, Nan Xu

**Affiliations:** Department of Hepatobiliary-Pancreatic-Splenic Surgery, Chengdu First People’s Hospital (Chengdu Integrated TCM & Western Medicine Hospital), High Tech District, No. 18 Wanxiang North Road, Chengdu, 610044 Sichuan People’s Republic of China

**Keywords:** Surgery, Therapeutic endoscopy

## Abstract

Common bile duct (CBD) exploration and T-tube drainage are the main surgical methods for the removal of bile duct stones (BDSs), which can now be completed by laparoscopy. However, the feasibility and safety of primary closure of the CBD (PCCBD) in laparoscopic CBD exploration (LCBDE) without biliary drainage are still uncertain. From January 1, 2021, to June 30, 2022, patients who were diagnosed with BDSs and underwent LCBDE and primary closure of the CBD without biliary drainage in our hospital were included. The clinical and prognostic data of the patients were retrospectively analyzed to determine the feasibility and safety of PCCBD in LCBDE without biliary drainage. Forty-nine patients successfully underwent PCCBD in LCBDE without biliary drainage. The operation time was 158.8 ± 50.3 (90–315,150) minutes, the bile duct suture time was 17.6 ± 4.46 (10–26, 18) minutes, the intraoperative blood loss volume was 70.4 ± 52.6 (5–200, 80) ml, the hospitalization cost was 28,141.2 ± 7011.3 (15,005.45–52,959.34, 26,815.14) CNY Yuan, the hospitalization time was 13.22 ± 5.16 (8–32, 12) days, and the postoperative hospitalization time was 7.31 ± 1.94 (3–15, 7) days. There were 3 cases of postoperative bile leakage (3/49, 6.12%), all of them healed by nonsurgical treatment. During the follow-up of 17.2 ± 11.01 (10–26, 17) months, no residual BDSs, biliary stricture or other complications classified as Clavien-Dindo grade I or higher occurred. For some selected patients who meet certain criteria, PCCBD in LCBDE without biliary drainage is feasible and safe and is more conducive to the rapid postoperative recovery of patients.

## Introduction

The management strategy for bile duct stones (BDSs) is mainly surgical or endoscopic stone removal. Surgical procedures mainly include open surgery and laparoscopic surgery, while endoscopic treatment is represented by endoscopic retrograde cholangiopancreatography (ERCP). Different treatment plans have their own indications, technical requirements, and advantages and disadvantages. Laparoscopic surgery and ERCP are the main minimally invasive methods. The current situation is that more doctors and patients choose minimally invasive methods to treat BDSs, which is also an important trend in the future.

The traditional surgical treatment for BDSs is open surgery to allow common bile duct (CBD) exploration and lithotomy combined with simultaneous T-tube placement to support decompression and drainage, avoid or alleviate possible bile leakage and stricture, and retain an approach for choledochoscopy to address residual stones postoperatively. With additional technological developments, laparoscopic CBD exploration (LCBDE) has been developed, which reduces the trauma of patients and is beneficial for the rehabilitation process. However, this method also requires T-tubes, which are related to some negative results, such as decreased quality of life and increased risks of bile leakage, difficulties associated with nursing care, T-tube dislocation, bile loss, and repeated postoperative examinations^1–5^.

Another method is to perform ERCP and place the nasobiliary drainage tube or biliary stent preoperatively and intraoperatively for primary closure of the CBD (PCCBD). However, there are risks associated with ERCP^6,7^, such as intubation failure, stone removal failure, pancreatitis, bleeding, endoscopic sphincterotomy (EST) if necessary, duodenal perforation and other related complications, and there are additional requirements for equipment, personnel and technical level. For example, post-ERCP pancreatitis (PEP) is a common serious complication, with a morbidity rate ranging from 4 to 10%. In high-risk patients, the morbidity rate can reach 15%, and the mortality rate is 0.7%, resulting in high medical expenses. According to data from the United States, the average annual cost for treating PEP is estimated to reach 200 million US dollars^8^.

At present, LCBDE has been increasingly performed in the operating theatre, but PCCBD is performed without a T-tube^7^. The major advantage of PCCBD is that the T-tube is not retained, which avoids complications related to the T-tube. However, patients still need to undergo placement of the nasobiliary duct or biliary stent by ERCP preoperatively or intraoperatively to help the bile duct heal after PCCBD^9,10^. Both intraoperative and preoperative placement of biliary stents through ERCP have complications such as occlusion and migration, which may require repeat removal or replacement after surgery^11^. A second ERCP may be needed to remove the implanted biliary stent. This process still increases the risk of complications related to ERCP^6,7^.

LCBDE-PCCBD without biliary drainage (including T-tube, nasobiliary duct, biliary stent) is a more reasonable scheme in theory. However, LCBDE-PCCBD is challenging in that it requires advanced laparoscopic skills and anatomical precision, and the clinical results are unknown^12^. The optimal timing for LCBDE-PCCBD without biliary drainage is still unclear^7^. In recent years, some studies have elucidated the advantages of LCBDE-PCCBD compared to traditional T-tube drainage^13–17^, but more evidence is still needed to clarify whether the postoperative complications of LCBDE-PCCBD, such as biliary stricture, biliary leakage and residual stones, can be effectively controlled, thereby making them acceptable. Therefore, we designed a retrospective study to evaluate the feasibility and safety of LCBDE-PCCBD without biliary drainage.

## Materials and methods

### Study design

This was a retrospective cohort study to judge the feasibility and safety of LCBDE-PCCBD without biliary drainage. Patients who were diagnosed with BDSs in our hospital from January 1, 2021, to June 30, 2022, and underwent LCBDE-PCCBD without biliary drainage were included. An alternative to ERCP delayed LC was provided, but LCBDE-PCCBD was selected by all 49 patients. All LCBDE-PCCBD procedures without biliary drainage were performed by 6 attending surgeons who had more than 10 years of clinical experience and who completed at least 100 bile duct explorations independently and successfully. All patients and/or their guardians signed relevant written informed consent forms before surgery, which met the medical ethical requirements. The study was reviewed and approved by the Institutional Ethical Committee of Chengdu First People’s Hospital (Chengdu Integrated TCM & Western Medicine Hospital) (2022-YNYJ-018). Due to the retrospective design of the study, informed consent was waived by the Ethics Committee of Chengdu First People’s Hospital (Chengdu Integrated TCM & Western Medicine Hospital) for this study. This study has been registered in the Chinese clinical trial registry (ChiCTR2200063032). The whole study process was conducted in accordance with the tenets of the Declaration of Helsinki.

### Patients and data collection

The clinical prognostic data of the enrolled patients were collected and analyzed. All data were obtained from the medical records database of our hospital. The inclusion criteria were as follows: (1) patients with a clear diagnosis of BDSs; (2) patients who agreed to primary closure of the CBD; (3) patients with BDSs but no hepatobiliary malignancies; and (4) patients with detailed clinical data records for analysis. The exclusion criteria were as follows: (1) patients with hepatobiliary malignancies; (2) patients who underwent placement of a T-tube or nasobiliary duct; (3) patients with clinical data that could not be analyzed; and (4) patients who were lost to follow-up.

The main observation indexes included patient demographic data, preoperative and postoperative liver function results, intraoperative conditions, postoperative recovery process and clinical outcomes within the follow-up period after the operation.

The postoperative complications were judged according to the Clavien‒Dindo classification system for surgical complications^18^ and the standard classification for bile leakage^19^.

### Surgical technical details

The operation was performed according to the procedures described below, and the procedure may have been modified when necessary for difficult cholecystectomies. The basic principles to avoid iatrogenic bile duct injury (BDI) were followed^20^.

The patients were in the reverse Trendelenburg position. Carbon dioxide pneumoperitoneum with a pressure of 10–13 mmHg was established by puncturing the infraumbilical region after general anesthesia with tracheal intubation. The 4-trocars technique was routinely used for surgical operation, one trocar (10 mm) was placed infra-umbilical to serve as the laparoscopic observation hole, one trocar (10–12 mm) was placed infra xiphoid process to serve as the main operating hole, allowing choledochoscope entry, stone clearance and suturing of the choledochotomy, one trocar (5 mm) was placed at the right subcostal midline of the clavicle and one trocar (5 mm) was placed at the anterior axillary line to serve as an auxiliary operation hole.

First, it was necessary to separate abdominal and gallbladder adhesions, if any. Then, the Calot triangle was dissected to expose the cystic duct and the cystic artery, and the CBD was identified simultaneously. The cystic artery was clamped and disconnected. The cystic duct was clamped but not disconnected temporarily, which helped to prevent stones from falling into the CBD. The gallbladder was completely freed by electrocoagulation from the gallbladder bed to allow traction and subsequent operation. After identification of the CBD, the serosa and anterior wall of the CBD were carefully cut by using a low-power electrotome so as to avoid causing dissection. According to preoperative MRI-MRCP, the longitudinal incision in the anterior wall of the CBD needed to be approximately 6 mm to 10 mm or slightly larger than the stone diameter. The bleeding point could be electrocoagulated for hemostasis. BDSs were eliminated with the stone basket through the choledochoscope. Complete stone clearance was achieved when the duodenal papilla could be seen clearly and completely opening and closing with the change in biliary pressure caused by water injection through the choledochoscope, at least the intrahepatic secondary bile duct could be reached upward during choledochoscopy and there was no stone residue. At the same time, the judgment is made in combination with the preoperative MRI-MRCP results. After stones were completely eliminated, the whole layer of the CBD wall was sutured intermittently or continuously using 3-0 or 4-0 absorbable sutures (VICRYL Plus, Ethicon Inc. Somervill, New Jersey, USA), with a margin of approximately 1.0 mm and a needle pitch of approximately 1.5 mm. After the completion of suturing, if there is bile leakage, it can be repaired by placement of intermittent sutures at the leakage point. All stitches had at least 4 tied knots. Finally, the gallbladder was completely removed after disconnecting the cystic duct, with the clamp remaining on the stump of the cystic duct. According to the situation, if it is difficult to remove the gallbladder or stones from the abdominal cavity, the incision can be appropriately expanded, or they can be placed in a retrieval bag and crushed before removal. Before the end of the operation, a peritoneal cavity drainage tube was routinely placed in the gallbladder fossa. Due to limited access to equipment and resources in our hospital, none of the patients underwent intraoperative cholangiography (IOC).

### Follow-up

All patients were followed up in the outpatient department after discharge, and the follow-up ended on April 31, 2023. At least one liver function test and ultrasound scan (USS) of the hepatobiliary system was completed during the follow-up period. MRI-MRCP was adopted if necessary. Whether the patients would receive subsequent treatment was determined according to the review results. In patients with residual or recurrent BDSs, ERCP was performed, and reoperation was necessary if ERCP failed. For patients with postoperative biliary stricture, biliary stent implantation is the first treatment choice. For patients whose liver function and imaging examinations were normal without subjective discomfort during the follow-up period, no more subsequent inspections were conducted.

### Statistical analysis

Continuous variables are presented as the mean ± SD (range, median), and categorical variables are presented as frequencies and percentages. The comparison of rates was based on counting data χ^2^ test. Mean number comparisons between the groups were performed by variance analysis. The mean number of paired data points was compared by t test. Statistical analyses were performed using SPSS 19 (IBM Corp., Armonk, NY, United States). A two-sided P value < 0.05 was considered statistically significant.

## Results

### Study cohort

A total of 49 eligible patients were enrolled, including 35 females and 14 males, with an average age of 53.37 ± 16.43 (17–83, 55) years. Sixteen patients had BDSs complicated by obstructive jaundice according to the upper limit of total bilirubin reference value used in our hospital (28 μmol/L). Twenty-three patients had varying degrees of acute cholecystitis, and 24 patients had varying degrees of acute cholangitis, as judged by the 2018 Tokyo Guidelines^21,22^. However, none of them had severe organ dysfunction (Tokyo Guidelines 2018-Grade III). All patients were confirmed to have CBDSs but no intrahepatic stones by preoperative MRI-MRCP. None of the patients underwent ERCP during this hospitalization period. Detailed preoperative clinical data are shown in Table 1.

**Table 1:** Preoperative clinical data of the patients who underwent LCBDE-PCCBD without biliary drainage (N = 49) (n, %; mean ± SD, range and median).

Female/male	35/14 (71.4/28.6)
Emergency/outpatient	20/29 (40.8/59.2)
Obstructive jaundice^a^ yes/no	16/33 (32.7/67.3)
Total bilirubin (μmol/L)	37.0 ± 48.9 (6.9–289.9, 19.2)
Direct bilirubin(μmol/L)	23.5 ± 41.4 (0.9–220.9, 6.3)
Alanine aminotransferase (U/L)	204.1 ± 230.3 (12–1039, 117)
Aspartate aminotransferase (U/L)	149.4 ± 178.6 (17–723, 47)
Albumin (g/L)	38.1 ± 3.59 (27.4–44.8, 38.1)
Gamma glutamyl transferase (U/L)	385.6 ± 388.2 (13–1830, 268)
Alkaline phosphatase (U/L)	162.6 ± 100.1 (51–460, 132)
Intrahepatic stones yes/no	0/49
Severity grade of acute cholecystitis No/1/2	26/16/7 (53.06/32.65/14.29)
Severity grade of acute cholangitis No/1/2	25/23/1 (51.02/46.94/2.04)
Comorbidity yes/no	21^b^/28 (42.9/57.1)
BMI	23.28 ± 3.20 (17.7–33.2, 23.1)
Age (yrs)	53.37 ± 16.43 (17–83, 55)
ASA 2/3	17/32 (34.7/65.3)
History time (m)	11.84 ± 28.09 (1–180, 1)
Number of previous visits	1.65 ± 1.14 (0–5, 2)
Previous surgery history yes/no	18^c^/31(36.7/63.3)

^a^According to the upper limit of total bilirubin reference value used in our hospital (28 μmol/L); ^b^mainly including pulmonary disease, hypertension and diabetes; ^c^including 4 cholecystectomies, 1 LC + ERCPs and 1 unsuccessful ERCP after previous cholecystectomy, the remaining procedures were nonhepatobiliary surgeries.

ASA: American Society of Anesthesiologists; BMI: body mass index.

### Intraoperative data

The intraoperative data are summarized in Table 2. Among the 49 patients who underwent LCBDE-PCCBD, 28 underwent continuous suture of the CBD (42.9%), and 28 underwent intermittent suture (57.1%), without a statistically significant difference in liver function between the two groups. Forty-three patients had varying degrees of difficulty for LC according to the literature^23^.

**Table 2:** Intraoperative data of LCBDE-PSCBD without biliary drainage (N = 49) (n, %; mean ± SD, range and median).

	Overall	Continuous suture n = 21	Intermittent suture n = 28	Statistics
DGS for LC: No/2/3/4	6/6/31/6	0/2/16/3	6/4/15/3	χ^2^ = 5.818, P = 0.121
Operative duration (min)	159 ± 50 (90–315,150)	170 ± 56 (112–315, 155)	151 ± 45 (90–260, 147.5)	*F* = 1.726, *P* = 0.195
Suture time (min)	18 ± 4.5 (10–26, 18)	15 ± 3.8 (10–20, 15)	19 ± 4.2 (15–26, 19)	*F* = 10.801, *P* = 0.002
Blood loss (ml)	70 ± 53 (5–200, 80)	59 ± 41 (5–150, 70)	79 ± 59 (10–200, 85)	*F* = 1.742, *P* = 0.193
Estimated CBD diameter (mm)	12.1 ± 3.3 (8–22, 11)	12.1 ± 3.7 (8–22, 11)	12.1 ± 3.0 (8–20, 12)	*F* = 0.006 *P* = 0.941
Estimated Stone diameter (mm)	8.3 ± 4.4 (3–20, 8)	6.7 ± 3.9 (3–17, 6)	9.5 ± 4.4 (3–20,8.5)	*F* = 0.021 *P* = 0.941
Number of stones (1/2/ ≥ 3)	27/13/9	12/5/4	15/8/5	χ2 = 5.078, P = 0.406

DGS: difficulty grading scale; LC: laparoscopic cholecystectomy; CBD: common bile duct.

### Postoperative and follow-up data

There were 3 cases of postoperative Grade A bile leakage (3/49, 6.12%), all of which were without preoperative jaundice (χ^2^ = 1.410, P = 0.325), all three had CBD wall sutured intermittently (χ^2^ = 2.397, P = 0.178); there were no statistically significant differences. In the patients with bile leakage, the abdominal drainage time was extended to 9–11 days and nutritional support and albumin infusion were provided when necessary. After the stoppage of bile leakage, the abdominal drainage tube was removed. There were 3 cases of hypoalbuminemia after the operation, which may have been caused by preoperative fasting due to pain, but all of them resolved after human albumin infusion and moderate nutritional support. No patient needed ICU treatment postoperatively. No malignant lesions were found in postoperative pathology for any of the patients.

The follow-up time for 49 patients after discharge was 17.2 ± 11.01 (10–26, 17) months. At the end of follow-up, no postoperative infection, postoperative hemorrhage, stone residue or recurrence, 30-day readmission or biliary stricture occurred, and no other postoperative complications classified as grade I or higher according to the Clavien‒Dindo classification occurred. The detailed postoperative follow-up data are shown in Table 3.

**Table 3:** Postoperative and follow-up outcomes of LCBDE-PSCBD without biliary drainage (N = 49) (n, %; mean ± SD, range and median).

Cost (CNY Yuan)	28,141.2 ± 7011.3 (15,005.45–52,959.34, 26,815.14)
Total hospital time (d.)	13.2 ± 5.2 (8–32, 12)
Preoperative hospital time (d.)	5.9 ± 4.3 (2–23, 4)
Postoperative hospital time (d.)	7.3 ± 1.9 (3–15, 7)
Antibiotic (d.)	9.2 ± 4.2 (0–24, 9)
Preoperative antibiotic (d.)	3.7 ± 3.2 (0–16, 3)
Postoperative antibiotic (d.)	5.5 ± 2.4 (0–14, 6)
Resume eating time (d.)	3.2 ± 1.3 (2–7, 3)
Gastric tube yes/no and time (d.)	11/38 (22.4/77.6); 1.3 ± 0.5 (1–2, 1)
Urinary catheter yes/no and time (d.)	39/10 (79.6/20.4); 1.9 ± 1.0 (1–5, 2)
Abdominal drainage yes/no and time (d.)	49/0 (100/0); 5.5 ± 1.8 (2–11, 5)
Postoperative infection	0
Postoperative hemorrhage	0
Hypoalbuminemia	3 (6.12)
Postoperative biliary leakage	3 (6.12)
Follow-up time (mo.)	17.2 ± 11.01 (10–26, 17)
Stone residue or recurrence	0
30-day readmission	0
Complications classified as Clavien-Dindo grade I or higher	0
Biliary stricture	0
ICU treatment	0
Malignant lesions	0

Overall, liver function was comparable to the preoperative level 1 week after surgery. By the end of the follow-up period, liver function had generally recovered and was significantly better than that before the operation and better than that 1 week after the operation.

## Discussion

Minimally invasive surgery for cholecystolithiasis and BDSs has been widely accepted and performed. Traditional open cholecystectomy, CBD exploration and stone removal, and T-tube drainage are currently not appropriate choices for most patients. ERCP combined with simultaneous or delayed LC or LC-LCBDE and T-tube drainage is a better treatment scheme.

However, ERCP has inherent defects^6,7^. At the same time, it is still necessary to undergo simultaneous or delayed LC under general anesthesia as means of complete treatment for cholecystolithiasis and CBDSs. Moreover, ERCP has some equipment, facility, personnel and technical requirements. As a result, ERCP cannot be the preferred solution for most patients, especially in countries and regions where medical resources are scarce.

LC-LCBDE and T-tube drainage were the products of the progress of surgical technology and instruments, making them the first treatment choices for most patients or a salvage plan when ERCP failure occurs. Compared with open surgery, laparoscopy has obvious advantages, such as its minimal invasiveness, but the T-tube must still be retained for a long time postoperatively, which may lead to negative results including diminished quality of life, increased difficulties in nursing care for patients with T-tubes or even T-tube dislocation, bile loss and electrolyte disorder, delayed recovery, and difficulty returning to normal work and social life. In particular, T-tube dislocation may require reoperation. The overall complication rate related to T-tubes ranges from 13.8% (open surgery) to 15.5% (laparoscopic surgery)^2^. More importantly, this complication rate is not related to open surgery or laparoscopic surgery but is related to the in-dwelling of the T-tube itself and the extraction process. The incidence of bile leakage after extubation was 10%, of which the incidence of biliary peritonitis was 54%, the mortality rate was 14%, and local scar healing of the biliary tract after extubation in some patients may lead to refractory long-term biliary stricture^5^. Wills et al.^3^ and Maghsoudi et al.^4^ independently reported that 274 and 1375 patients underwent CBD exploration with T-tube placement, and the final complication rate and mortality rate related to T-tubes were 15.3% (42/274) and 0.73% (2/274) and 2.47% (34/1375) and 5.9% (2/34), respectively. Therefore, T-tube-related complications were an important reason for prolonged hospitalization, increased reoperation rate and higher medical costs, even leading to death.

In recent years, PCCBD in LCBDE has been used in the operating theatre to avoid the disadvantages of the above treatment scheme. However, many patients still need to undergo ERCP to allow placement of a nasobiliary tube or biliary stent preoperatively or intraoperatively^9,10,12,24^, the main purpose of which is to decompress the CBD and reduce the incidence of postoperative biliary leakage. However, this scheme involving ERCP still needs to meet the requirements for facilities, equipment and personnel, and ERCP-related complications might occur. The nasobiliary tube still needed to be preserved for some time, resulting in bile loss.

Some scholars have reported on the surgical method of primary closure of CBD (PCCBD) in LCBDE without biliary drainage (including T-tube, nasobiliary tube and biliary stent). Its advantage is that almost all the disadvantages of the abovementioned surgical scheme are avoided without increasing the incidence of complications. Tan et al.^25^ reported the clinical data of 27 patients who underwent LCBDE-PCCBD without biliary drainage. The median length of the operation was 160 min (80–265), the average diameter of the CBD was 14.5 mm (7–30 mm), the maximum diameter of the stones was 1.2 cm (0.3–2.6 cm), the intraoperative blood loss volume was 30 ml (10–50), and there was 1 case of postoperative bile leakage with residual stones (3.7%). Yang et al.^26^ reported that 81 patients underwent LCBDE-PCCBD without biliary drainage, 32 patients (39.5%) underwent intermittent suture of the biliary tract, 49 patients (60.5%) underwent continuous suture, the average operative time was 123 min, and the average intraoperative blood loss volume was approximately 40 ml. There were 2 patients (2.5%) with grade A and B biliary leakage after the operation, and 1 patient (1.23%) received follow-up ERCP treatment for grade B bile leakage. Biliary bleeding was stopped by octreotide + hemostatic treatment, and 4 patients with postoperative cholangitis needed antibiotic treatment. In addition, there was 1 case of pneumonia and 1 case of acute pancreatitis requiring medical treatment. Zhan et al.^27^ reported the data for 408 LCBDE-PCCBD procedures. The average diameter of the CBD was approximately 12 mm, there were 12 cases (2.94%) of postoperative bile leakage, 1 case (0.25%) of biliary stricture, 1 case (0.25%) of residual stone, 1 case (0.25%) of 30-day readmission, 1 case (0.25%) of stone recurrence, and 3 cases (0.74%) of reoperation.

Compared with the abovementioned studies, our study presented similar results. All 49 patients successfully underwent LCBDE-PCCBD without biliary drainage. From the intraoperative data, both intermittent suture and continuous suture was safe and effective, without increasing the operation time or blood loss volume. Only 3 cases of postoperative Grade A bile leakage occurred, all of which were cured by nonsurgical treatment. No other complications classified as grade I or higher according to the Clavien-Dindo classification system occurred. Overall, liver function recovered well after the operation. In particular, no biliary stricture or residual or recurrent stones occurred postoperatively or during the follow-up period.

According to the above results of our study, we propose the following surgical indications for LCBDE-PCCBD: (1) the diameter of the CBD is not less than 8 mm; (2) there is no malignant tumor of the bile duct; (3) there is no grade III acute cholangitis or grade III acute cholecystitis; (4) there are no residual stones found during the operation; and (5) there is no biliary stricture. Through intraoperative choledochoscopy and preoperative MRI-MRCP results to comprehensively judge whether there are residual stones, intraoperative cholangiography may be recommended if necessary. Moreover, previous biliary and upper abdominal surgery does not constitute a contraindication for LCBDE-PCCBD. The difficulty in grading for LC does not affect the progress of LCBDE-PCCBD. Combined with hypertension, diabetes or COPD, LCBDE-PCCBD can also be performed safely on the premise of adequate preoperative preparation. The above indication criteria may help to select the patients who are most suitable for LCBDE-PCCBD.

In addition, LCBDE-PCCBD does not require a separate learning curve, as long as experienced doctors who are skilled in conventional LCBDE can successfully complete LCBDE-PCCBD in patients who meet the above surgical indications.

Transcystic duct exploration (TCDE) is an alternative surgical strategy, but it is not routinely performed. Its success rate varies greatly among different reports and is influenced by various factors, such as stone diameter, location and angle of the cystic duct entering the CBD. At the same time, TCDE may still require partial incision of the CBD or dilation of the cystic duct, which may lead to cystic duct rupture, laceration or avulsion and may require more special equipment, such as a 3 mm choledochoscope^28^. Therefore, we did not perform TCDE in these patients in our study.

Our present study was limited in that data retrieved from a single institution were retrospectively analyzed, and the included sample size was not large enough. The findings need to be further confirmed in a prospective study with a larger sample size and a longer follow-up period.

## Conclusion

LCBDE-PCCBD without biliary drainage is feasible and safe among the selected patients who meet certain criteria. Such a procedure is conducive to the postoperative recovery of patients, and the postoperative complication rate can be controlled in a relatively low range.

## Data Availability

The database used and/or analyzed during the current study is not publicly available but is available from the corresponding author upon reasonable request.
